# Evaluation of Self-Etching Adhesive and Er:YAG Laser Conditioning on the Shear Bond Strength of Orthodontic Brackets

**DOI:** 10.1155/2013/719182

**Published:** 2013-10-08

**Authors:** Rosalía Contreras-Bulnes, Rogelio J. Scougall-Vilchis, Laura E. Rodríguez-Vilchis, Claudia Centeno-Pedraza, Oscar F. Olea-Mejía, María del Carmen Z. Alcántara-Galena

**Affiliations:** ^1^Universidad Autónoma del Estado de México, Facultad de Odontología, Centro de Investigación y Estudios Avanzados en Odontología (CIEAO), Jesús Carranza Esquina Paseo Tollocan, Colonia Universidad, 50130 Toluca, MEX, Mexico; ^2^Universidad Autónoma del Estado de México Universidad Nacional Autónoma de México, Centro Conjunto de Investigación en Química Sustentable (CCIQS), Km 14.5 Carretera Toluca-Ixtlahuaca, San Cayetano de Morelos, 50200 Toluca, MEX, Mexico

## Abstract

The purpose of this study was to evaluate the shear bond strength, the adhesive remnant index scores, and etch surface of teeth prepared for orthodontic bracket bonding with self-etching primer and Er:YAG laser conditioning. One hundred and twenty bovine incisors were randomly divided into four groups. In Group I (Control), the teeth were conditioned with 35% phosphoric acid for 15 seconds. In Group II the teeth were conditioned with Transbond Plus SEP (5 sec); III and IV were irradiated with the Er:YAG 150 mJ (11.0 J/cm^2^), 150 mJ (19.1 J/cm^2^), respectively, at 7–12 Hz with water spray. After surface preparation, upper central incisor stainless steel brackets were bonded with Transbond Plus Color Change Adhesive. The teeth were stored in water at 37°C for 24 hours and shear bond strengths were measured, and adhesive remnant index (ARI) was determined. The conditioned surface was observed under a scanning electron microscope. One-way ANOVA and chi-square test were used. Group I showed the significantly highest values of bond strength with a mean value of 8.2 megapascals (MPa). The lesser amount of adhesive remnant was found in Group III. The results of this study suggest that Er:YAG laser irradiation could not be an option for enamel conditioning.

## 1. Introduction

Surface conditioning is essential to increase bond strength to enamel. The direct bonding of orthodontic brackets with composite resins has been considered as one of the most significant developments in orthodontics [1]. The enamel etching technique has been accepted as a routine procedure for bonding orthodontic brackets to the tooth surfaces [2]. However, a potential disadvantage is the possibility of decalcification, which leaves the enamel susceptible to caries attack, especially under orthodontic attachments [3–5]. There is a need to simplify clinical procedures and minimize enamel loss while maintaining clinically useful bond strength [6, 7]. In an effort to improve the adhesion procedure, reduce loss of enamel, prevent saliva contamination, and save chairside time, self-etching primers (SEPs) have been introduced in the market [8, 9].

The use of Er:YAG laser for caries removal and cavity preparation was approved in 1997 by the US Food and Drug Administration (FDA) [10]. Although this laser was introduced into dentistry for the ablation of dental hard tissue [11, 12], laser sub ablation has become available as an alternative to acid etching of enamel and also dentine [13]. Research on enamel surface roughness showed that laser irradiation yielded a comparable or similar amount of surface roughness as that seen with acid-etch [14]. It has been reported that laser etching inhibits caries, and this could be of great importance in orthodontics [15, 16]. Because water spraying and air drying are not needed with laser etching, procedural errors can be reduced and time saved. Therefore, laser irradiation might be a suitable technique to etch enamel for orthodontic bonding.

The purpose of this study was to evaluate the shear bond strength, the adhesive remnant index scores, and etching surface of teeth prepared for orthodontic bracket bonding with Er:YAG laser etching, phosphoric acid etching, and a self-etching primer.

## 2. Materials and Methods

### 2.1. Sample Selection and Storage

A total of 120 freshly extracted bovine teeth were strictly selected with intact buccal surfaces following the criteria described by Bishara et al. [17]. The collected teeth were stored in a solution of 0.2 percent (wt/vol) thymol until used, during a period no longer than two months.

### 2.2. Sample Preparation

The teeth were cleansed and pumiced using a rubber cup with fluoride-free paste for 10 seconds, after which they were thoroughly washed with water and air dried. Then, teeth were fixed in acrylic resin (Orthodontic Resin, Dentsply Caulk International Inc., USA), with a label bearing the number of each sample. A mounting jig was used to align the labial surface of the tooth to be perpendicular to the bottom of the mold and its buccal surface parallel to the force during subsequent bond strength testing.

The teeth were randomly assigned to one of the four groups; enamel conditioning protocols are shown in Table 1.

*Group I: Phosphoric Acid. *The acid gel was applied on the enamel for 15 sec, rinsed thoroughly with a forceful air-deionized water spray, and dried with compressed air.

*Group II: Self-Etching Primer. *The SEP was activated according to manufacturer's instructions. The resulting mix was then applied by continuously rubbing on the enamel surface for 5 sec. The SEP was then lightly dried using compressed air for 1 to 2 sec.

*Groups III and IV: Er:YAG Laser Conditioning. *Energy levels were calibrated with the calipers of the equipment, and the energy delivered was measured periodically with a power meter (LaserMate-P, Coherent Co., Santa Clara, CA, USA). The irradiation was manually performed in one direction such that the tips were smoothly scanned only once, perpendicular to the enamel surface of the samples. In both irradiated groups, the fixed parameters were the wavelength at 2.94 *μ*m, energy pulse 150 mJ, pulse duration 250–400 *μ*sec, deionized water spraying at 5.0 mL/min, total scanning time of irradiated area 15 sec, and tip-sample distance fixed at 1 mm. At that tip-sample distance, the exit tip and the laser beam had the same diameter, as corroborated with a laminated infrared sensor screen (Lumitek International, Inc., Ijamsville, MD, USA). After Er:YAG laser irradiation, the samples were rinsed thoroughly with deionized water spray and dried with compressed air.

### 2.3. Brackets

The stainless steel brackets used were 0.018-inch Gemini (3M, Unitek, Monrovia, CA, USA) for upper central incisor. The average surface area of the bracket base was determined to be 12.62 mm^2^. This value was obtained by randomly measuring 10 bracket bases.

### 2.4. Bonding Procedure

Prior to bonding, Transbond MIP (3M Unitek) was applied in Groups I, III, and IV according to the manufacturer's instructions. In all groups the brackets were bonded with Transbond Plus Color Change (3M Unitek). The bonding adhesive was light-cured with Ortholux Luminous Curing Light (3M Unitek) for a total of 20 seconds.

### 2.5. Storage

A 0.019 × 0.025 inch stainless steel wire was ligated into each bracket slot to reduce any deformation of the bracket during the debonding process. The teeth were fixed in acrylic resin, and a mounting jig was used to align the facial surface of the tooth so that it was parallel to the force during the SBS test. Afterwards, the teeth were stored in distilled water at 37°C for 24 hours.

### 2.6. Shear Bond Strength (SBS) Test

Bond strengths were then measured at a crosshead speed of 0.5 mm/min, and the force applied at the time of fracture was recorded in newtons (N) and converted to megapascals (Mpa) dividing the force by the bracket base area.

### 2.7. Adhesive Remnant Index (ARI)

Once the brackets had been debonded, the enamel surface of each tooth was examined with a stereoscope (Nikon, Tokyo, Japan) at a magnification of 10x to determine the amount of residual adhesive remaining on each tooth. The ARI scores were recorded as described by Årtun and Bergland, 1984 [18], with the following scale used: 0 = no adhesive left on the tooth, 1 = less than half of the adhesive left on the tooth, 2 = more than half of the adhesive left on the tooth, and 3 = all adhesive left on the tooth, with distinct impression of the button mesh.

### 2.8. Scanning Electron Microscopy (SEM) Observation of Enamel Surface

After the etching procedure, one sample from each group was dried and attached to a testing ring with adhesive carbon paper (SPI Supplies, West Chester, PA, USA) for observation under a scanning electron microscope (SEM) (JEOL, JSM-6510LV, Tokyo, Japan).

### 2.9. Statistical Analysis

Descriptive statistics including the mean, standard deviation, and Scheffé multiple comparisons (one-way ANOVA) with significance predetermined at *P* < 0.05 were calculated for the SBS analysis. In addition, the chi-square test was used to evaluate the ARI.

## 3. Results

The SBS, expressed in MPa, and descriptive statistics are shown in Table 2; laser groups showed the lowest ranges.

The ARI scores indicating the amount of adhesive remaining after debonding are shown in Table 3. Chi-square comparison of the ARI scores amongst all groups (*χ* ^2^ = 116.377) indicated that the groups were significantly different (*P* = 0.000). The least amount of adhesive remnant was found in Group III.

**Figure 1:**
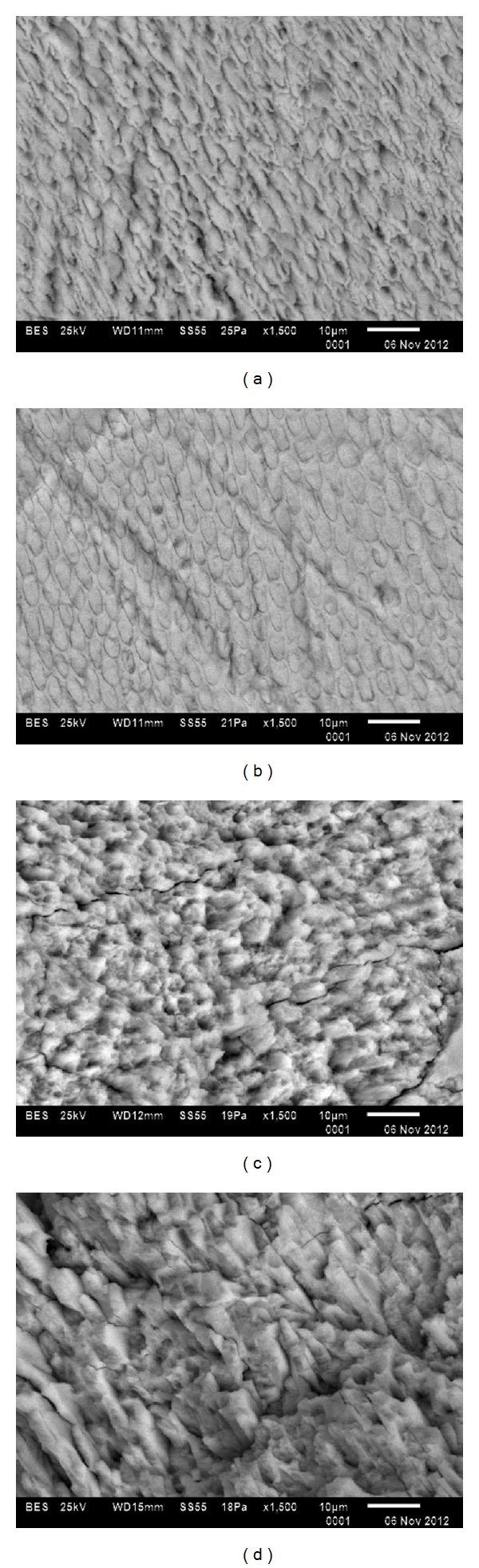
SEM micrographs of enamel etching patterns by group. (a) Etched for 15 sec with 35% phosphoric acid (Group I). (b) Conditioned with SEP Transbond Plus (Group II). (c) Enamel irradiated with Er:YAG laser at 11.0 J/cm^2^ (Group III). (d) Enamel irradiated with Er:YAG laser at 19.1 J/cm^2^ (Group IV).

SEM analysis showed the different etching patterns obtained for each group (Figure 1). After phosphoric acid treatment (Figure 1(a)), a typical etching pattern was observed, mixed prism centers and prism periphery etching. Self-etching group (Figure 1(b)) showed an etching pattern less pronounced, with an apparent lower loss of interprismatic substance. For Er:YAG laser etching groups (Figures 1(c) and 1(d)) a rough surface with an increased exposure along the prisms was observed, as well as microcracks.

## 4. Discussion

Technological innovations as well as the evolution of dental materials for the conditioning of enamel offer the clinician several options for orthodontic brackets bonding. In this study, we evaluated three enamel conditioning techniques for bracket bonding in terms of SBS, ARI, and SEM observations. The most common conditioning technique used in bonding procedures was used as control group (phosphoric acid), whereas it is the method that has proved to be the most effective in terms of shear bond strength. However, acid etching has been associated with decalcification and a greater degree of enamel loss [19–21].

The bond strength value for the self etching primer group was comparable to that obtained for the control group. Additionally, SEM micrographs showed a more conservative conditioning surface with a lower extent of damage to enamel than that produced by phosphoric acid. Furthermore, it has been reported that SEPs prevent saliva contamination and save chairside time [8].

Concerning Er:YAG laser groups, bonding force was similar among them. Nevertheless, conditioning pattern in the enamel surface was specific for each group. According to SEM observations, a lower retention shown by Er:YAG laser conditioning could be explained due to smooth areas on the conditioned enamel surface, being a possible effect of the laser pulse. We recommend additional research to evaluate other scanning techniques for Er:YAG laser conditioning, in order to achieve a homogeneous surface that improves bond strength.

A rougher surface was achieved at the irradiation conditions used in Group IV, associated with a higher energy density and bond strength, without statistically significant difference in comparison to self-etching primer group. However, a clinically acceptable bonding value from 5.9 to 7.8 MPa [22] was not reached.

Although there are previous reports in the literature related to the Er:YAG conditioning of enamel for orthodontic bonding [23–29], it is difficult to compare them because of diverse conditions employed for irradiation. Reports agree that Er:YAG laser conditioning is a method potentially adequate for orthodontic bonding [23–27], but inferior to that obtained after conventional acid etching [24, 29], as shown in this study. Other authors [23–25] have reported that a combination of Er:YAG laser conditioning and acid etching produces the best retention. However, this method could result in substantial loss of enamel tissue.

## 5. Conclusions

The results of this study suggest that Er:YAG laser irradiation is not an option for enamel conditioning, under the experimental parameters employed.

## Figures and Tables

**Table 1:** Conditioning for control and experimental groups.

	Groups			
	I (Control) Phosphoric acid	II Self-Etching Primer (SEP)	III Er:YAG laser	IV Er:YAG laser
Enamel conditioning	Ultra-Etch, Ultradent Products, Inc.	Transbond Plus SEP, 3M Unitek	OPUS DUO Er:YAG + CO_2_, Lumenis	
	Concentration: 35% Time: 15 sec	Time: 5 sec	Frequency: 7 Hz Sapphire tip *Ø*: 1.3 mm Energy density: 11.0 J/cm^2^	Frequency: 12 Hz Sapphire tip *Ø*: 1.0 mm Energy density: 19.1 J/cm^2^

**Table 2:** Mean bond strength values (MPa) and descriptive statistics.

Group	n	Mean	SD	Range	Sheffé*
I	30	8.2	4.3	6.6–9.8	A
II	30	6.8	2.8	5.7–7.8	A, B
III	30	2.9	1.7	2.2–3.5	C
IV	30	4.8	2.1	4.0–5.6	B, C

*Groups with different letters are significantly different (*P* = 0.000) from each other.

**Table 3:** Distribution frequency and percentages of the adhesive remnant index (ARI).

Group	ARI scores (%)				n
	0	1	2	3	
I	1 (3.3)	5 (16.6)	16 (53.3)	8 (26.6)	30
II	1 (3.3)	11 (36.6)	9 (30)	9 (30)	30
III	29 (96.6)	1 (3.3)	0 (0)	0 (0)	30
IV	5 (16.6)	20 (66.6)	3 (9.9)	2 (6.6)	30

*χ* ^2^ = 116.377; df = 9; *P* = 0.000.

## References

[1] D’Attilio M, Traini T, Di Iorio D, Varvara G, Festa F, Tecco S (2005). Shear bond strength, bond failure, and scanning electron microscopy analysis of a new flowable composite for orthodontic use. *Angle Orthodontist*.

[2] Buonocore MG (1955). A simple method of increasing the adhesion of acrylic filling materials to enamel surfaces. *Journal of Dental Research*.

[3] Gorelick L, Geiger AM, Gwinnett AJ (1982). Incidence of white spot formation after bonding and banding. *American Journal of Orthodontics*.

[4] Sudjalim TR, Woods MG, Manton DJ, Reynolds EC (2007). Prevention of demineralization around orthodontic brackets *in vitro*. *American Journal of Orthodontics and Dentofacial Orthopedics*.

[5] Øgaard B, Rølla G, Arends J (1988). Orthodontic appliances and enamel demineralization. Part 1. Lesion development. *American Journal of Orthodontics and Dentofacial Orthopedics*.

[6] Bishara SE, VonWald L, Laffoon JF, Warren JJ (2001). Effect of a self-etch primer/adhesive on the shear bond strength of orthodontic brackets. *American Journal of Orthodontics and Dentofacial Orthopedics*.

[7] Yamada R, Hayakawa T, Kasai K (2002). Effect of using self-etching primer for bonding orthodontic brackets. *Angle Orthodontist*.

[8] Vicente A, Bravo LA, Romero M (2005). Influence of a nonrinse conditioner on the bond strength of brackets bonded with a resin adhesive system. *Angle Orthodontist*.

[9] Tecco S, Traini T, Caputi S, Festa F, De Luca V, D’Attilio M (2005). A new one-step dental flowable composite for orthodontic use: an *in vitro* bond strength study. *Angle Orthodontist*.

[10] Sulewski JG (2000). Historical survey of laser dentistry. *Dental Clinics of North America*.

[11] Hibst R, Keller U (1989). Experimental studies of the application of the Er:YAG laser on dental hard substances: I. Measurement of the ablation rate. *Lasers in Surgery and Medicine*.

[12] Keller U, Hibst R (1989). Experimental studies of the application of the Er:YAG laser on dental hard substances: II. Light microscopic and SEM investigations. *Lasers in Surgery and Medicine*.

[13] Walsh LJ, Abood D, Brockhurst PJ (1994). Bonding of resin composite to carbon dioxide laser-modified human enamel. *Dental Materials*.

[14] Hess JA (1990). Scanning electron microscopic study of laser-induced morphologic changes of a coated enamel surface. *Lasers in Surgery and Medicine*.

[15] Klein ALL, Rodrigues LKA, Eduardo CP, Dos Santos MN, Cury JA (2005). Caries inhibition around composite restorations by pulsed carbon dioxide laser application. *European Journal of Oral Sciences*.

[16] Üşümez S, Orhan M, Üşümez A (2002). Laser etching of enamel for direct bonding with an Er,Cr:YSGG hydrokinetic laser system. *American Journal of Orthodontics and Dentofacial Orthopedics*.

[17] Bishara SE, Soliman M, Laffoon J, Warren JJ (2005). Effect of antimicrobial monomer-containing adhesive on shear bond strength of orthodontic brackets. *Angle Orthodontist*.

[18] Årtun J, Bergland S (1984). Clinical trials with crystal growth conditioning as an alternative to acid-etch enamel pretreatment. *American Journal of Orthodontics*.

[19] Bishara SE, VonWald L, Laffoon JF, Jakobsen JR (2000). Effect of altering the type of enamel conditioner on the shear bond strength of a resin-reinforced glass ionomer adhesive. *American Journal of Orthodontics and Dentofacial Orthopedics*.

[20] van Waes H, Matter T, Krejci I (1997). Three-dimensional measurement of enamel loss caused by bonding and debonding of orthodontic brackets. *American Journal of Orthodontics and Dentofacial Orthopedics*.

[21] Wickwire NA, Rentz D (1973). Enamel pretreatment: a critical variable in direct bonding systems. *American Journal of Orthodontics*.

[22] Reynolds IR (1975). A review direct orthodontic bonding. *British Journal of Orthodontics*.

[23] Türköz Ç, Ulusoy Ç (2012). Evaluation of different enamel conditioning techniques for orthodontic bonding. *Korean Journal of Orthodontics*.

[24] Lasmar MF, Reher VG, Lalloo R, Reher P (2012). Enamel demineralization and bracket bond strength when etching with acid and /or Er:YAG laser. *Australian Dental Journal*.

[25] Basaran G, Hamamci N, Akkurt A (2011). Shear bond strength of bonding to enamel with different laser irradiation distances. *Lasers in Medical Science*.

[26] Gokcelik A, Ozel Y, Ozel E (2007). The influence of Er:YAG laser conditioning versus self-etching adhesives with acid etching on the shear bond strength of orthodontic brackets. *Photomedicine and Laser Surgery*.

[27] Kim J-H, Kwon O-W, Kim H-I, Kwon YH (2005). Effectiveness of an Er:YAG laser in etching the enamel surface for orthodontic bracket retention. *Dental Materials Journal*.

[28] Lee B-S, Hsieh T-T, Lee Y-L (2003). Bond strengths of orthodontic bracket after acid-etched, Er:YAG laser-irradiated and combined treatment on enamel surface. *Angle Orthodontist*.

[29] Martínez-Insua A, Dominguez LDS, Rivera FG, Santana-Penín UA (2000). Differences in bonding to acid-etched or Er:YAG-laser-treated enamel and dentin surfaces. *Journal of Prosthetic Dentistry*.

